# Identification of Levels of Serum Amyloid A and Apolipoprotein A1 in Serum Proteomic Analysis of Neuropsychiatric Systemic Lupus Erythematosus Patients

**DOI:** 10.1155/2018/6728541

**Published:** 2018-11-21

**Authors:** Nancy P. Duarte-Delgado, Tania P. Lujan, Álvaro Arbeláez-Cortés, Jenny García-Valencia, Adriana Zapata, Mauricio Rojas, Mauricio Restrepo-Escobar, Gloria Vásquez, Blanca L. Ortiz-Reyes

**Affiliations:** ^1^Universidad Manuela Beltrán, Departamento de Ciencias Básicas, Bogotá, Colombia; ^2^Hospital San Rafael, Medicina General, Itagüí, Colombia; ^3^Clínica de Artritis Temprana S.A.S., Reumatología, Cali, Colombia; ^4^Universidad de Antioquia, Facultad de Medicina, Grupo Académico en Epidemiología Clínica (GRAEPIC), Medellín, Colombia; ^5^Clínica de Salud Mental Integral S.A.S.-SAMEIN, Medellín, Colombia; ^6^Universidad de Antioquia, Facultad de Medicina, Grupo de Inmunología Celular e Inmunogenética (GICIG), Medellín, Colombia; ^7^Universidad de Antioquia, Facultad de Medicina, Grupo de Reumatología Universidad de Antioquia (GRUA), Medellín, Colombia

## Abstract

Neuropsychiatric Systemic Lupus Erythematosus (NPSLE) has multiple pathogenic mechanisms that cause diverse manifestations and whose diagnosis is challenging because of the absence of appropriate diagnostic tests. In the present study the application of proteomics using two-dimensional electrophoresis (2D) and mass spectrometry (MS) allowed the comparison of the protein profile of the serum low and high abundance protein fractions of NPSLE patients (NPSLE group) and SLE without neuropsychiatric syndromes (SLE group), Neuropsychiatric syndromes not associated with SLE (NPnoSLE groups), and healthy controls (CTRL group). The gels obtained were digitalized and analyzed with the PDQuest software. The statistical analysis of the spots was performed using the nonparametric Kruskal Wallis and Dunn's multiple comparison tests. Two spots showed significant differences and were identified by MS. Spot 4009 was significantly lower in NPSLE with regard to NPnoSLE (p= 0,004) and was identified as apolipoprotein A1 (APOA1) (score 809-1132). Spot 8001 was significantly higher in NPSLE regarding CTRL and NPnoSLE (p= 0,01 y 0,03, respectively) and was identified as serum amyloid A (SAA) (score 725-2488). The proinflammatory high density lipoproteins (HDL) have been described in SLE. In this HDL the decrease of APOA1 is followed by an increase in SAA. This altered level of both proteins may be related to the inflammatory state that is characteristic of an autoimmune disease like SLE, but this is not specific for NPSLE.

## 1. Introduction

Systemic Lupus Erythematosus (SLE) is a chronic autoimmune disease caused by the loss of immune tolerance to nuclear and cytoplasmic autoantigens in genetically susceptible individuals exposed to environmental triggers. The production of autoantibodies allows the formation of immune complexes, whose deposition in several body organs causes inflammation and tissue damage [1, 2].

Neuropsychiatric Systemic Lupus Erythematosus (NPSLE) comprises 19 syndromes described by the American College of Rheumatology (ACR) [3]. There is a great variability in the prevalence reported for NPSLE syndromes, which ranges from 12% to 95% for different studies [4]. That inconsistency has fallen upon the inclusion of minor and nonspecific symptoms (such as depression, headaches, and cognitive dysfunction) which may appear under several circumstances [5]. There is also a failure in the attribution of neuropsychiatric symptoms to SLE (known as primary NPSLE) or as a result of nervous system injuries indirectly involved with SLE (secondary NPSLE) [6]. This makes the correct attribution of neuropsychiatric syndromes to SLE dependent on an exclusion process, because of the absence of a diagnostic gold standard useful for these manifestations [7].

The lack of a confirmatory test for NPSLE is due to the multiple mechanisms that cause its diverse manifestations. The study of these pathogenic mechanisms has allowed proposing some biomarkers for NPSLE. IL-6 is a cytokine that has shown a strong association with NPSLE and it has been involved with neuronal damage [8–10]. It has been shown that the TWEAK/Fn14 signaling pathway promotes the disruption of the blood-brain barrier and increases cell neuronal death associated with NPSLE [11]. The immune complexes present in the cerebrospinal fluid (CSF) are potent amplifiers of inflammation in the brain inducing IFN*α*, CXCL10, CCL-2, and IL-18, which are cytokines and chemokines that have been found elevated in the CSF of NPSLE patients [12]. There was reported an increase in MMP-9 levels of NPSLE patients compared with SLE patients without neuropsychiatric involvement and healthy controls, which is directly related to the potential of this inflammatory mediator of damaging brain parenchyma [13, 14].

The “omics” disciplines including proteomics have offered the development of disease biomarkers that improve the diagnostic, prognostic, surveillance, and treatment of several diseases [15]. Since research has demonstrated that serum protein levels reflects human physiologic states [16], its proteomic analysis has become an alternative for the search of potential biomarkers, as it allows the identification and relative quantification of proteins [17].

In the present study the application of proteomics using two-dimensional electrophoresis (2D) and mass spectrometry (MS) allowed the comparison of the protein profile of the low and high abundance protein fractions, of serum samples of NPSLE patients (NPSLE group) and SLE without neuropsychiatric syndromes (SLE group), neuropsychiatric syndromes not associated with SLE (NPnoSLE group), and healthy controls (HC group). It was found that apolipoprotein A1 (APOA1) was decreased, while serum amyloid A (SAA) was increased in these patients regarding those presenting NPnoSLE and/or CTRL groups.

## 2. Materials and Methods

### 2.1. Materials

Acrylamide/bis solution 40%, ReadyStrip™ IPG (Immobilized pH gradient) strips 17 cm and pH 4-7, TEMED (tetramethylenediamine), ammonium persulfate (APS), 2-mercaptoethanol, iodoacetamide, equilibration buffer I and II, mineral oil, overlay agarose,* Precision Plus Protein*™* Dual Color Standards, Precision Plus Protein*™* Dual Xtra Prestained Protein Standards*, Aurum™* Serum Protein Mini Kit *are from BioRad (Hercules, CA, USA). Phosphate Buffered Saline (PBS)is from Gibco (Grand Island, NY, USA). Glycine, acetone, and ethanol are from Fisher Scientific (Hampton, NH, USA). Acetonitrile (ACN) and hydrochloric acid (HCl) are from Merck Millipore (Frankfurt, Germany). Glycerol molecular biology grade is from Promega (Madison, WI, USA). SDS (sodium dodecyl sulphate) and trifluoroacetic acid (TFA) are from Sigma–Aldrich (St Louis, MO, USA). Ultrapure Urea, ZOOM Thiourea, Ultrapure Tris, ZOOM CHAPS (3-(3-Cholamidopropyl)dimethylammonio-1-propanesulfonate), dithiothreitol (DTT), and SilverQuest™ Silver Staining Kit are from Thermo Fischer Scientific (Waltham, MA, USA).

### 2.2. Patients and Samples

All the subjects that participated in the study were at least 18 years old. The written informed consent was obtained from all the participants before their inclusion in the study. The study included eighteen patients with active SLE that had been hospitalized between the years 2012 and 2014 in Hospital Universitario San Vicente Fundación (HUSVF). These patients were diagnosed according to the 1982 revised criteria for the classification of SLE [18]. The SLE group included nine patients that did not have neuropsychiatric syndromes. The NPSLE group included nine patients whose neuropsychiatric symptoms were associated with SLE disease activity (primary NPSLE) that were classified following the guidelines given by the nomenclature and case definitions for NPSLE [3]. The NPnoSLE group included nine patients with a neuropsychiatric syndrome not associated with SLE, infections, metabolic alterations, trauma, and/ or tumors that assisted in medical consultation at Clínica de Salud Mental Integral SAMEIN S.A.S. because they recently presented a psychotic episode and that were diagnosed using the Diagnostic and Statistical Manual of Mental Disorders (DSM IV). These samples were obtained between July and August 2015. The CTRL group was conformed by nine students and teachers from Sede de Investigación Universitaria de la Universidad de Antioquia whose ages matched with those corresponding to the NPSLE group. Their samples were also collected in the time lapse between 2012 and 2014. Pregnancy was an exclusion criterion for all groups.

The clinical information regarding prescribed medication and disease indexes (SLEDAI (Systemic Lupus Erythematosus Disease Activity Index) for SLE patients [19], SAPS and SANS (Scale for the Assessment of Positive and Negative Symptoms, respectively) for NPnoSLE patients [20]), and the demographic characteristics from the patients (sex and age) were obtained from the medical records. This study was approved by Comité Central de Ética en la Investigación (CCEI) from Universidad de Antioquia and HUSVF. The blood samples for the research were collected into red cap Vacutainer blood collection tubes.

### 2.3. Serum Processing

The serum protein concentration was quantified using the Pierce™ BCA Protein Assay Kit. This procedure allowed determining the concentration of serum sample to load the columns of the Aurum™ Serum Protein Mini Kit, to generate the enrichment of two different protein fractions: the high and low abundance protein fractions. The high abundance protein fraction corresponded to those proteins that remain attached to the gel matrix of the column and is represented mainly by albumin and IgG (which are the most abundant proteins of serum), although there were many other proteins present. On the other hand, those proteins that flow through the columns constituted the low abundance protein fraction. The high abundance proteins were extracted from the gel matrix using an extraction buffer (5 M urea, 2 M thiourea, 2% CHAPS, and 2% ASB 14) and then were precipitated adding ice cold acetone overnight. The samples obtained for both fractions were dialyzed to remove salts and other small compounds that might have interfered with the subsequent procedures. Once the processing was complete the samples were quantified, and their integrity was assessed through SDS-PAGE. Finally, they were stored at -70°C.

### 2.4. Two-Dimensional Electrophoresis (2D)

For two-dimensional electrophoresis (2D) the IPG strips (17 cm and pH 4-7) were passively rehydrated during 16 h with 300 *μ*L hydration buffer (8M urea, 2% CHAPS, 50mM DTT, 0,2% ampholytes 3-10, and 0,001% bromophenol blue) containing 10 *μ*g of the high abundance proteins or 15 *μ*g of the low abundance proteins. The first dimension was run in the PROTREAN i12 IEF system using the steps reported by Cubedo et al. in 2011: (1) linear step at 250 V for 45 min; (2) linear step at 500 V for 1 h; (3) linear step at 1000 V for 1 h; (4) linear step at 4000 V for 1 h; (5) linear step at 10000 V for 1 h; (6) linear step at 10000 V until reaching 63000 V [21]. The proteins in the strips were reduced with equilibration buffer (375 mM Tris HCl pH 8.8, 6 M urea, and 2% SDS) and 2% DTT. Then, they were alkylated with the equilibration buffer containing 2.5% iodoacetamide. Fort the second dimension the strips were attached to a 12% polyacrylamide gel (dimensions 20 x 20 cm) using 0,5% agarose. The proteins were run using the buffer MES SDS 1X in the PROTEAN® xi cell, applying 100 V for 30 minutes and 150 V for 12 h at 4°C. The recommendations of Westermeier and Naven were followed for the improvement of the resolution of the 2D [22]. The gels were stained with the SilverQuest™ Silver Staining Kit.

### 2.5. Image Acquisition and Data Analysis

The images were obtained in the Gel Doc™ XR+ System using a white light conversion screen. The software that allowed image visualization was the Image Lab™. The images were saved in the format.TIFF with a depth of 16 bits, for their further analysis in the software PDQuest. The detection of the spots was automatically performed by setting the following parameters: sensitivity, minimum peak value, size scale, background intensity, and streak removal. Once the spots were detected, the original images were filtered and smoothed to clarify the spots. This allowed PDQuest to determine which spots were differentially expressed in NPSLE regarding each one of the other study groups (NPnoSLE, SLE, and CTRL). In order to assess if the differential expression of the spots selected was statistically significant the data intensity was analyzed with the software GraphPad Prism 6 using the Kruskal Wallis nonparametric test, followed by Dunn's multiple comparisons. The comparisons were considered significant if they had a p-value less than 0,05. The fold change of these spots was calculated to determine the rate of increase or decrease of the intensity of the NPSLE spots compared with the other study groups.

### 2.6. Protein Identification by Mass Spectrometry (MS)

For the identification of the proteins present in the spots of interest, they were excised and sent to the Bindley Bioscience Center at Purdue University. The proteins were digested using 0,05 *μ*g/*μ*L trypsin and then were extracted from the gels using a solution containing 60% ACN and 5% TFA. The samples were run in the Eksigent 425 Nano LC system coupled with the triple TOF 5600+ mass spectrometry system from Sciex. The MS data obtained was further analyzed using the software Mascot Daemon v 2.5.1 to obtain the protein scores through the comparison between the peptides masses experimentally obtained with those founds in the Uniprot-Human data base. Carbamidomethylation was set as a fixed modification, whereas oxidation was set as a variable modification. Fragment mass tolerance was 0,2 and peptide mass tolerance was 0,05. Only one missed cleavage was allowed, and the significance threshold ranged from 0,01 to 0,02.

## 3. Results

### 3.1. Study Groups and Clinical Characteristics of the Patients

The patients that participated in the study were nine for each of the four study groups (NPSLE, SLE, NPnoSLE, and HC). The patients from the SLE group were all women and the median of the SLEDAI was 12 (Table 1). To be classified as active SLE patients the SLEDAI score had to be greater than or equal to 4. This group presented criteria for SLE classification like the following: arthritis, kidney disease, hematologic disease, proteinuria, and malar rash. They presented the autoantibodies ANAs and anti-DNA (data not shown). The patients from the NPSLE group were also all women and the median of the SLEDAI was 22 (Table 1). Four of them had psychosis, two had seizure disorders, one presented both psychosis and seizures, one had headaches, and others had acute confusional state. They presented a larger diversity of autoantibodies besides ANAs and anti-DNA, like anti-RNP, anti-Ro, anti-LA, anti-Sm, and anti-cardiolipin IgG (data not shown). The patients of the NPnoSLE group were mostly men that recently had gone through a psychotic episode. The scales used to evaluate schizophrenia symptoms were SANS and SAPS. The median of the SANS was 42, while for SAPS it was 18,5 (Table 1). The characteristics of the individuals included in the study groups are described in Table 1.

### 3.2. Differential Expression Analysis of Proteins

**Figure 1:**
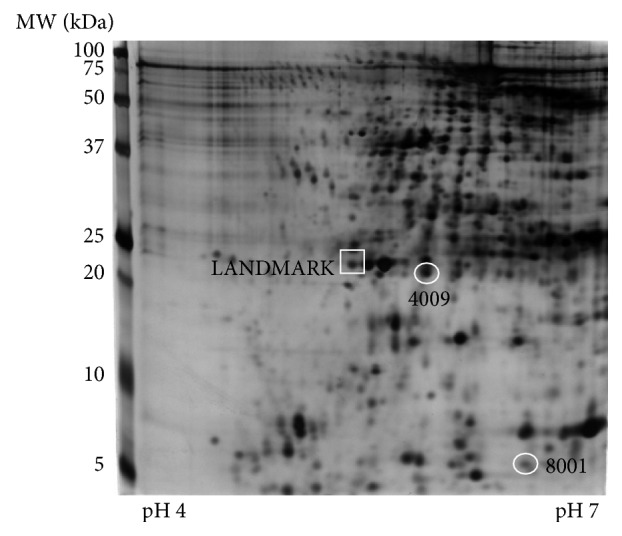
**Two-dimensional gel electrophoresis image showing the two protein spots that were consistently increased or decreased in NPSLE patients compared to the other groups.** The encircled spots resulted differentially expressed in the high abundance fraction. The calculated molecular weights (MW) and isoelectric points for spot 4009 were 20,9 kDa and pH = 5,9, respectively and for spot 8001 were 6,1 kDa and pH =6,5. The square shows the landmark used for matching 2D images for the comparative analysis. MW: molecular weight in kDa.

The differential expression analysis of proteins resulted in two spots selected because their abundance resulted significantly different in NPSLE group with respect to NPnoSLE group. The spots were named 4009 and 8001 and are shown in Figure 1. Both were discovered analyzing the high abundance protein fraction, which remarks the potential of this serum protein fraction that has been ignored in the past. These spots correspond to low molecular weight proteins since they have less than 25 kDa. The spot 4009 had a calculated molecular weight of 20,9 kDa and the isoelectric point was pH 5,9, whereas for the spot 8001 the values obtained were 6,1 kDa and pH 6,5, respectively. The landmark corresponds to a reference spot that is useful in making the correct alignment between the gels, during the differential expression analysis in the software.

**Figure 2:**
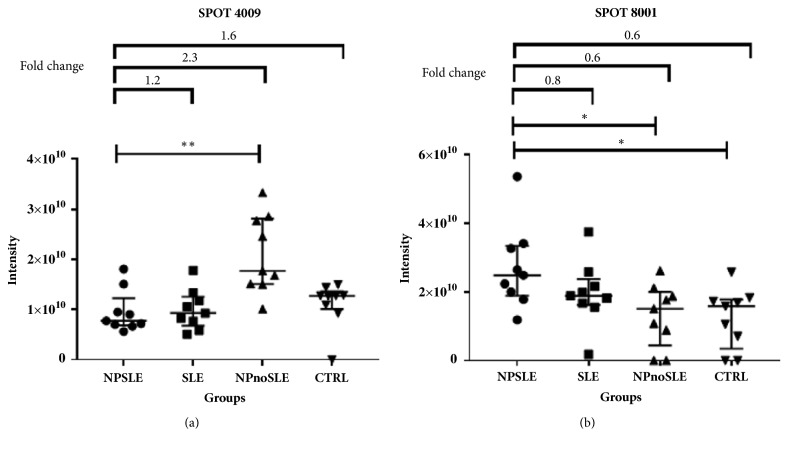
**Intensity of spots 4009 (a) and 8001 (b) in NPSLE patients compared with SLE, NPnoSLE, and CTRL groups. **The median and IQR of the intensities are shown for each group in both spots. These spots resulted differentially expressed in NPSLE with respect to NPnoSLE group in the case of spot 4009 (a) and also to the CTRL group for spot 8001 (b). The statistical analysis included the nonparametric Kruskal-Wallis test as well as Dunn's test for the multiple comparisons. *∗* p≤ 0,05 and *∗∗* p≤ 0,01. The fold change values are shown in the upper side of the figure.

The spot 4009 was significantly decreased in NPSLE with regard to NPnoSLE (p= 0,004). It was also evident a fold decrease of 2,3 of this spot in NPSLE comparing with NPnoSLE (Figure 2(a)). On the other hand, the spot 8001 was significantly increased in NPSLE regarding HC and NPnoSLE groups (p= 0,001 and p= 0,03, respectively) (Figure 2(b)).

### 3.3. Identification of Spots

Once the differential expression of the spots 4009 and 8001 was determined, they were excised and the proteins present were identified by LC-MS/MS. According to the results obtained, the spot 4009 corresponds to apolipoprotein A1 (APOA1), since it had the largest protein score (score range 809-1132) among the proteins reported. Regarding the analysis of spot 8001, it resulted that the only listed protein that obtained a good score (score range 725-2488) was serum amyloid A (SAA) (see Table 2). Considering the results of the differential expression analysis, it can be determined that there is a decrease of APOA1 (spot 4009) and an increase of SAA (spot 8001) levels in NPSLE with respect to the NPnoSLE and/or HC groups.

## 4. Discussion 

The present study used proteomics as the biomarker discovery approach that allowed the detection of two serum proteins that are differentially expressed in serum of patients with NPSLE with respect to NPnoSLE and/or CTRL groups: APOA1(spot 4009) and SAA (spot 8001) (Figure 2). Both proteins are part of the apolipoproteins present in plasma HDL (high density lipoproteins). HDL are responsible for maintaining the equilibrium in the plasma concentration of cholesterol and triglycerides through their removal from the systemic circulation towards the liver, in a process called reverse cholesterol transport [23].

APOA1 is the most abundant apolipoprotein from HDL and it is responsible for many of their functions, including reverse cholesterol transport [24]. This apolipoprotein has also an immunomodulatory role in the catchment and neutralization of lipopolysaccharide, which diminishes the severity of the tissue damage it causes [25]; it also participates in the inhibition of different proinflammatory pathways through the regulation in the composition of the cell membrane lipids of immune cells [26, 27]. On the other hand, SAA is a protein produced by the liver as a result of the acute phase response [28]. This consists in a systemic reaction of the organism against disturbances caused by infections, tissue damage, trauma or surgery, neoplastic growth, or immunological disorders [29]. Since SAA appears during acute phase reaction it is known as an acute phase protein.

APOA1 and SAA are related proteins that change their expression during the acute phase reaction. The SAA produced by hepatocytes during acute phase reaction is released into blood circulation, where it associates with HDL. A marked increase in plasma SAA levels can displace APOA1, transforming HDL in proinflammatory molecules [24, 28]. The results obtained in this study are in concordance with this brief explanation of the relationship between APOA1 and SAA, because the greater increase of SAA was observed in the NPSLE group, where APO1 might have been replaced by SAA in HDL.

The proinflammatory HDLs have been described in SLE patients. They are also characterized by decreased content in protective proteins such as APOA1 and increased levels of the prooxidant SAA. The features that make this HDL proinflammatory are that they enhance the oxidation of LDL and therefore the activation of immune cells like monocytes, anti-oxLDL antibody production, and immune complex formation [24]. As previously stated, proinflammatory HDL have increased levels of SAA, which are also evident in the joints of patients with rheumatoid arthritis, in the blood vessels with atherosclerotic lesions, and in many types of solid tumors [28]. There have been reported autoantibodies against APOA1 in SLE patients, which may be related to the decrease in this apolipoprotein [30].

One study showed that APOA1 suppresses proinflammatory signaling induced by CD40 in macrophages through the removal of cholesterol from the lipid rafts, which prevents translocation of CD40 to the adapting molecule TRAF-6 in the cell membrane. The decrease in APOA1 may activate the production of many proinflammatory cytokines like IL-6 and IL-8 [31], while the increase in SAA induces the production of IL-1*β* and IL-23 in monocytes [28].

## 5. Conclusion

The decrease in APOA1 and the increase in SAA showed specifically in NPSLE in this study may be related to the inflammatory state that is characteristic of an autoimmune disease like SLE. This means that the variation of these two proteins depends on immunological features and that is the reason why they allow distinguishing the SLE patients with neuropsychiatric manifestations, from those that have neuropathologies not associated with SLE. However, it is necessary to continue with studies to understand the relationship of APOA1 and SAA with the physiopathological mechanisms involved with NPSLE, to continue elucidating their possible role as biomarkers.

## Figures and Tables

**Table 1:** **Study groups and clinical characteristics of the patients.** The study groups were the following: NPSLE (Neuropsychiatric Systemic Lupus Erythematosus), SLE (Systemic Lupus Erythematosus), NPnoSLE (Neuropsychiatric syndromes not associated with SLE), and CTRL (healthy controls). The characteristics compared among the groups were the median and IQR of the age (in years), sex (female or male), and the median and IQR of the SLEDAI, SANS, and SAPS scales. SLEDAI*∗* = Systemic Lupus Erythematosus Disease Activity Index; SANS*∗∗* = Scale for the Assessment of Negative Symptoms; SAPS*∗∗∗* = Scale for the Assessment of Positive Symptoms.

GROUP	N°	AGE (YEARS)	SEX (F/M)	SLEDAI*∗*, SANS*∗∗* and SAPS*∗∗∗*
		MEDIAN; IQR		MEDIAN; IQR
NPSLE	9	29 y; 31	9/0	SLEDAI 22; 10,5

SLE	9	27 y; 18,5	9/0	SLEDAI 12; 11,5

NPnoSLE	9	30 y; 24	3/6	SANS 42; 44,25
				SAPS 18,5; 23,25

CTRL	9	24 y; 20,5	9/0	N/A

**Table 2:** **Proteins of interest identified by LC-MS/MS.** The MASCOT server performed the match analysis and the search of proteins using Uniprot_Human database. According to that, spot 4009 is apolipoprotein A1 and 8001 is serum amyloid A.

**Spot**	**Accession number**	**Protein name**	**Score range**
4009	A0A024R3E3_HUMAN	Apolipoprotein A1	809 – 1132

8001	D3DQX7_HUMAN	Serum Amyloid A	725 – 2488

## Data Availability

The data used to support the findings of this study are available from the corresponding author upon request.

## References

[1] Liu Z., Davidson A. (2012). Taming lupus-a new understanding of pathogenesis is leading to clinical advances. *Nature Medicine*.

[2] Yu S.-L., Kuan W.-P., Wong C.-K. (2012). Immunopathological roles of cytokines, chemokines, signaling molecules, and pattern-recognition receptors in systemic lupus erythematosus. *Journal of Immunology Research*.

[3] Anon (1999). The American College of Rheumatology nomenclature and case definitions for neuropsychiatric lupus syndromes. *Arthritis & Rheumatism*.

[4] Unterman A., Nolte J. E. S., Boaz M., Abady M., Shoenfeld Y., Zandman-Goddard G. (2011). Neuropsychiatric syndromes in systemic lupus erythematosus: a meta-analysis. *Seminars in Arthritis and Rheumatism*.

[5] Kampylafka E. I., Alexopoulos H., Kosmidis M. L. (2013). Incidence and Prevalence of Major Central Nervous System Involvement in Systemic Lupus Erythematosus: A 3-Year Prospective Study of 370 Patients. *PLoS ONE*.

[6] Hanly J. G., Urowitz M. B., Su L. (2010). Prospective analysis of neuropsychiatric events in an international disease inception cohort of patients with systemic lupus erythematosus. *Ann. Rheum. Dis*.

[7] Hanly J. G., Harrison M. J. (2005). Management of neuropsychiatric lupus. *Best Practice & Research Clinical Rheumatology*.

[8] Hirohata S., Kanai Y., Mitsuo A., Tokano Y., Hashimoto H. (2009). Accuracy of cerebrospinal fluid IL-6 testing for diagnosis of lupus psychosis. A multicenter retrospective study. *Clinical Rheumatology*.

[9] Fragoso-Loyo H., Richaud-Patin Y., Orozco-Narváez A. (2007). Interleukin-6 and chemokines in the neuropsychiatric manifestations of systemic lupus erythematosus. *Arthritis & Rheumatology*.

[10] Trysberg E., Nylen K., Rosengren L. E., Tarkowski A. (2003). Neuronal and Astrocytic Damage in Systemic Lupus Erythematosus Patients with Central Nervous System Involvement. *Arthritis & Rheumatology*.

[11] Wen J., Doerner J., Weidenheim K. (2015). TNF-like weak inducer of apoptosis promotes blood brain barrier disruption and increases neuronal cell death in MRL/lpr mice. *Journal of Autoimmunity*.

[12] Santer D. M., Yoshio T., Minota S. (2009). Potent induction of IFN-alpha and chemokines by autoantibodies in the cerebrospinal fluid of patients with neuropsychiatric lupus. *J. Immunol*.

[13] Trysberg E., Blennow K., Zachrisson O., Tarkowski A. (2004). Intrathecal levels of matrix metalloproteinases in systemic lupus erythematosus with central nervous system engagement.. *Arthritis research & therapy*.

[14] Ainiala H., Hietaharju A., Dastidar P. (2004). Increased serum matrix metalloproteinase 9 levels in systemic lupus erythematosus patients with neuropsychiatric manifestations and brain magnetic resonance imaging abnormalities. *Arthritis & Rheumatology*.

[15] Arriens C., Mohan C. (2013). Systemic lupus erythematosus diagnostics in the ‘omics’ era. *International Journal of Clinical Rheumatology*.

[16] Hu S., Loo J. A., Wong D. T. (2006). Human body fluid proteome analysis. *Proteomics*.

[17] Tirumalai R. S., Chan K. C., Prieto D. A., Issaq H. J., Conrads T. P., Veenstra T. D. (2003). Characterization of the Low Molecular Weight Human Serum Proteome. *Molecular & Cellular Proteomics*.

[18] Tan E. M., Cohen A. S., Fries J. F. (1982). The 1982 revised criteria for the classification of systemic lupus erythrematosus. *Arthritis & Rheumatology*.

[19] Bombardier C., Gladman D. D., Urowitz M. B., Caron D., Chang C. H. (1992). Derivation of the SLEDAI. A disease activity index for lupus patients. *Arthritis Rheum*.

[20] Andreasen N. C., Flaum M., Swayze V. W., Tyrrell G., Arndt S. (1990). Positive and negative symptoms in schizophrenia. A critical reappraisal. *Archives of General Psychiatry*.

[21] Cubedo J., Padró T., García-Moll X., Pintó X., Cinca J., Badimon L. (2011). Serum proteome in acute myocardial infarction. *Clínica e Investigación en Arteriosclerosis*.

[22] Westermeier R., Naven T. (2002). Troubleshooting in proteomics. *Proteomics in Practice: A laboratory Manual of Proteome Analysis*.

[23] Fonslow B. R., Stein B. D., Webb K. J., Xu T., Choi J., Kyu S. (2013). High Density Lipoprotein Biogenesis, Cholesterol Efflux, and Immune Cell Function. *Arterioscler. Thromb. Vasc. Biol*.

[24] von Eckardstein A., Kardassis D. (2015). Impact of Systemic Inflammation and Autoimmune Diseases on apoA-I and HDL Plasma Levels and Functions. *High Density Lipoproteins, Handbook of Experimental Pharmacology*.

[25] Ma J., Liao X., Lou B., Wu M., Gong Z. (2004). Role of Apolipoprotein A-I in Protecting against Endotoxin Toxicity. *Acta Biochimica et Biophysica Sinica*.

[26] Umemoto T., Han C. Y., Mitra P. (2013). Apolipoprotein AI and high-density lipoprotein have anti-inflammatory effects on adipocytes via cholesterol transporters: ATP-binding cassette A-1, ATP-binding cassette G-1, and scavenger receptor B-1. *Circulation Research*.

[27] Wang S.-H., Yuan S.-G., Peng D.-Q., Zhao S.-P. (2012). HDL and ApoA-I inhibit antigen presentation-mediated T cell activation by disrupting lipid rafts in antigen presenting cells. *Atherosclerosis*.

[28] Ye R. D, Sun L. (2015). Emerging functions of serum amyloid A in inflammation. *J. Leukoc. Biol*.

[29] Gruys E., Toussaint M. J. M., Niewold T. A., Koopmans S. J. (2005). Acute phase reaction and acute phase proteins. *Journal of Zhejiang University SCIENCE A*.

[30] Batuca J. R., Ames P. R. J., Amaral M., Favas C., Isenberg D. A., Delgado Alves J. (2009). Anti-atherogenic and anti-inflammatory properties of high-density lipoprotein are affected by specific antibodies in systemic lupus erythematosus. *Rheumatology*.

[31] Yin K., Chen W., Zhou Z. (2012). Apolipoprotein A-I Inhibits CD40 Proinflammatory Signaling via ATP-Binding Cassette Transporter A1-Mediated Modulation of Lipid Raft in Macrophages. *Journal of Atherosclerosis and Thrombosis*.

